# Evaluation of the Efficacy of a New Dichoptic Digital Platform to Treat the Anisometropic and Isometropic Amblyopia

**DOI:** 10.3390/brainsci12070815

**Published:** 2022-06-22

**Authors:** Md Oliullah Abdal, Faiza Bhombal, Gul J. Nankani, Sonia G. Nankani, Shruti Lad, Aditi Dholam, Richa Kumari, Jinal Mahajan, David P. Piñero

**Affiliations:** 1 Krishna Eye Centre, Mumbai 400012, India; abdaloliullah@bynocs.com (M.O.A.); faiza.bhombal@gmail.com (F.B.); drnankani@gmail.com (G.J.N.); nankanisonia@gmail.com (S.G.N.); shruti.lad15@gmail.com (S.L.); aditis14893@gmail.com (A.D.); richa.stardust@gmail.com (R.K.); jinalvshah27@gmail.com (J.M.); 2 Department of Optics, Pharmacology and Anatomy, University of Alicante, 03690 Alicante, Spain; 3 Department of Ophthalmology, Vithas Medimar International Hospital, 03016 Alicante, Spain

**Keywords:** amblyopia, anisometropia, dichoptic training, stereopsis

## Abstract

The aim of the current study was to evaluate the results of a novel dichoptic training program using an online platform in a group of subjects with refractive amblyopia, performing a comparative analysis of unilateral and bilateral amblyopic cases. For this purpose, a retrospective study analysis of data of 161 children (4–13 years) who underwent dichoptic treatment with the Bynocs^®^ platform (Kanohi Eye Pvt. Ltd., Mumbai, India) was performed. In all cases, the therapy protocol consisted of sessions of training of 30 min daily 5 times a week for 6 weeks. Best corrected visual acuity (BCVA) in the non-dominant eye improved significantly with the treatment, with a mean change of 0.39 logMAR in the whole sample (*p* < 0.001). Regarding binocularity, the binocular function (BF) score also experienced a significant improvement (*p* < 0.001), with a mean change of 1.55 with therapy in the whole sample. The BCVA of the dominant eye only improved significantly (*p* < 0.001) in the isometropic amblyopic subgroup. In conclusion, the use of the dichoptic therapy with the digital platform evaluated allows an effective restoration of visual acuity and binocular function in children with anisometropic and isometropic amblyopia.

## 1. Introduction

The treatment of amblyopia has changed significantly in last few years due to better knowledge of the neural mechanism of this condition [1] and the development of new therapies, some of them combined with videogames [2]. The interruption or weakening of binocular vision during childhood, due to factors such as strabismus or anisometropia leading to different levels of interocular suppression has a primary role in the development of amblyopia and consequently should be overcome considering the peculiarities of each case [3]. For this reason, a great variety of studies have been conducted to evaluate different approaches employing simultaneous and separate stimulation of both eyes, which is called dichoptic, to eliminate the interocular suppression and to improve the visual acuity in amblyopia [4,5]. However, the dichoptic environment is not the only aspect that should be considered in visual training in amblyopia as the selection of the type of stimuli to use is also crucial [6].

There is a great variety of studies confirming the benefit of dichoptic treatment in children with amblyopia after appropriate refractive correction, especially in anisometropic amblyopia [7,8,9,10,11,12,13,14,15,16,17,18,19]. However, there are a limited number of trials reporting the opposite, a lack of benefit of a dichoptic approach in amblyopia compared to placebo [20,21]. It should be considered that aspects such as the stimuli used, the compliance or the type of amblyopia are crucial aspects for a successful outcome of the dichoptic therapy [6,22]. As an example, dichoptic stimulation cannot be done in a strabismic patient without achieving and maintaining bifoveal fixation during the training (surgery, prisms). Likewise, as happened with patching [23], results of treatment of bilateral amblyopia with visual training seem to be more limited, although evidence in this type of case is still scarce [24].

The aim of the current study was to evaluate the results of a novel dichoptic training program using an online platform in a group of subjects with refractive amblyopia, performing a comparative analysis of unilateral and bilateral amblyopic cases.

## 2. Materials and Methods

### 2.1. Patients

This was a retrospective study analysing the data of 161 children who underwent Bynocs^®^ Amblyopia therapy (Kanohi Eye Pvt. Ltd., Mumbai, India) during the time period of January 2019 to January 2022. The research protocol followed the tenets of the Declaration of Helsinki.

Inclusion criteria were children with ages of 4–13 years diagnosed with anisometropic or isometropic amblyopia with bifoveal fixation, best corrected visual acuity (BCVA) between 0.2 LogMAR to 1.0 LogMAR in the amblyopic eye, BCVA of 0.1 LogMAR or better in the non-amblyopic eye, heterophoria below 5 prism diopters, assessed by the Bynocs diagnostic module on the Bynocs platform and treated with such a platform. Likewise, all children were required to have been wearing their appropriate correction glasses for at least 8 weeks before initiating the visual training. Exclusion criteria were myopia greater than 6.00 D, any amblyopia treatment such as patching, atropine penalisation, Bangerter filter, or vision therapy done in the previous 2 weeks, previous intraocular surgery, developmental delay or any other ocular complication other than amblyopia.

### 2.2. Clinical Protocol

A complete baseline ophthalmic examination was performed in all patients including manifest and cycloplegic refraction, measurement of BCVA with a LogMAR chart, fixation pattern evaluation by ophthalmoscope, distance and near stereoacuity testing with the Bynocs^®^ Randot Test, 4-dot Worth test and measurement of the heterophoria with the Bynocs program, anterior segment evaluation with slit lamp and thereafter dilated retinal examination with indirect ophthalmoscopy. At the end of the training program, BCVA, ocular alignment and stereoacuity were tested under the same conditions in order to confirm the improvement achieved.

### 2.3. Visual Training Protocol

**Figure 1:**
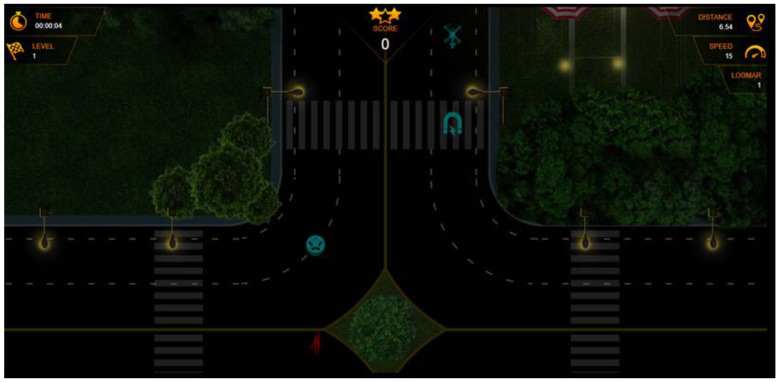
Example of a dichoptic environment used by the platform evaluated that is seen through red-green glasses (only one eye sees the red superman and the other one the rest of the elements).

The Bynocs^®^ Amblyopia therapy protocol consisted of sessions of training of 30 min daily 5 times a week for 6 weeks. The activities included dichoptic exercises as well as Fusional Vergence Exercises. Dichoptic exercises consisted of scenes that were mostly seen by the dominant eye (use of red-blue goggles for dissociation) while one crucial stimulus for the performance of the game was only seen by the non-dominant eye (Figure 1). The size of these stimuli could be modified in size according to the visual acuity of the patient.

### 2.4. Statistical Analysis

The primary outcome measure was the mean change in BCVA after 6 weeks of treatment and the secondary outcome measure was mean change in distance and near stereoacuity after 6 weeks. The Kolmogorov–Smirnov normality test was performed and the data were found not to be normally distributed. Nonparametric statistical tests were applied for the data analysis: Wilcoxon test to analyse the significance of changes pre-post and Mann–Whitney test to compare the outcomes between unilateral and bilateral amblyopia groups. The McNemar test was used to assess the significance of differences in percentages between the pre- and post-therapy visits. The Spearman correlation coefficient was calculated to evaluate the level of correlation between the visual change achieved and different baseline conditions.

For the analysis of the level of binocularity, the binocular function score (BF) was calculated, considering the following: value of 5 as suppression, value of 4 as simultaneous vision or flat fusion and from 1.6 to 3.3 (log 40 arc sec − log 2000 arc sec) as the presence of stereopsis [25].

## 3. Results

The sample evaluated included a total of 161 participants, with ages ranging from 4 to 13 years old (mean: 8.2; standard deviation: 2.5; median: 8.0 years). The distribution of the sample in terms of gender was as follows: 85 males (52.8%) and 76 females (47.2%). Out of 161 participants, 127 (78.9%) were diagnosed with anisometropic amblyopia and 34 (21.1%) with isometropic amblyopia. A total of 35 patients (21.7%) were previously treated with patching, all from the anisometropic amblyopia group.

### 3.1. Analysis of the Whole Sample

**Figure 2:**
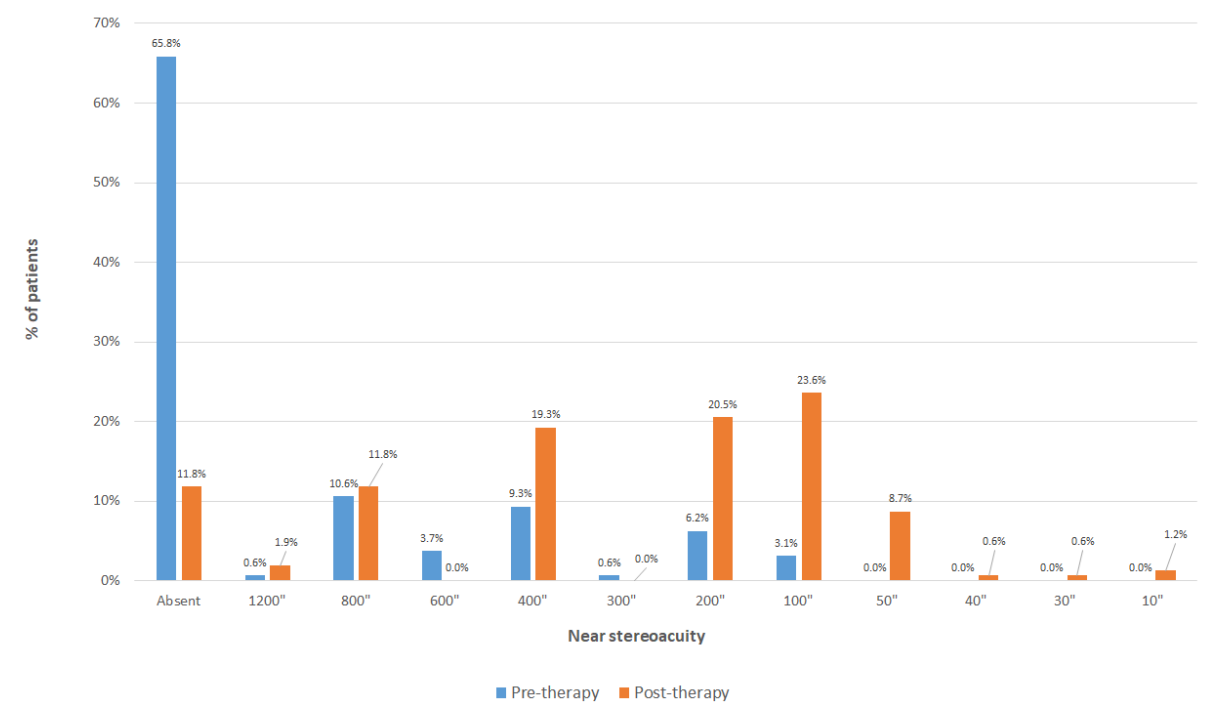
Distribution of the pre-and post-therapy measurements of near stereoacuity in the whole sample evaluated.

**Figure 3:**
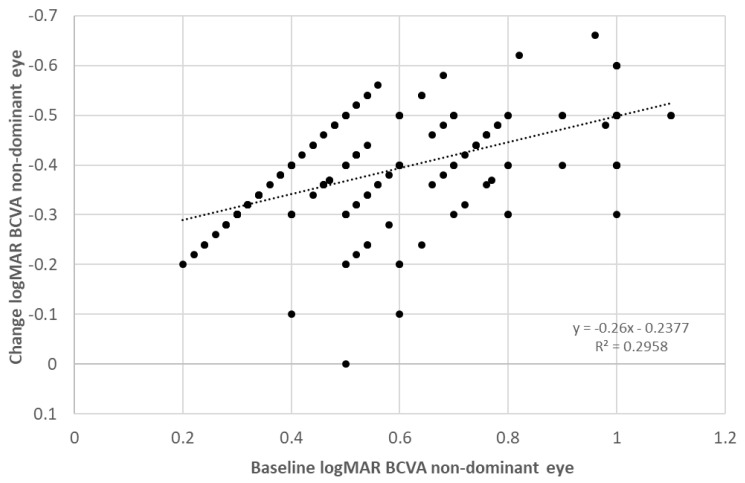
Scatter plot showing the relationship between the change in best corrected visual acuity (BCVA) in the non-dominant eye and the baseline BCVA in such eye. The adjusting line to the data obtained by means of the least-squares fit is shown.

**Figure 4:**
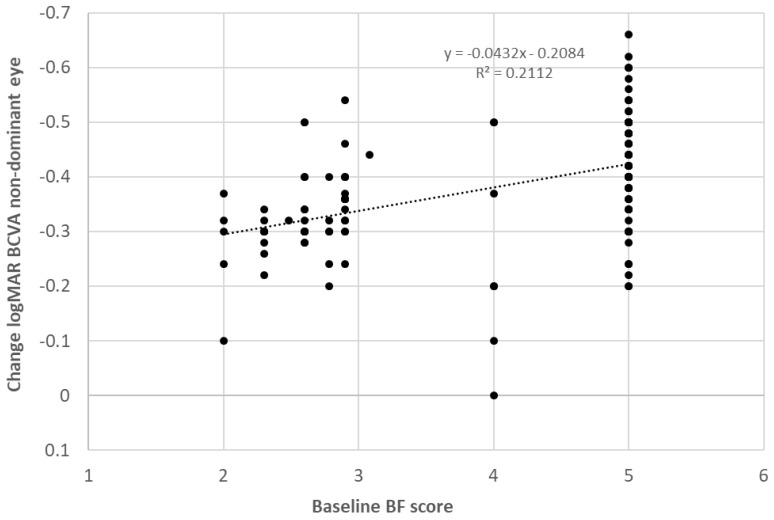
Scatter plot showing the relationship between the change in best corrected visual acuity (BCVA) in the non-dominant eye and the baseline binocular function (BF) score. The adjusting line to the data obtained by means of the least-squares fit is shown.

In the whole sample, BCVA in the non-dominant eye improved significantly with the treatment, with a mean change of 0.39 logMAR (almost 4 logMAR lines) (*p* < 0.001). Likewise, the BCVA of the dominant eye also experienced a significant improvement (*p* < 0.001), with a mean change of 0.08 logMAR (almost 1 logMAR line). Regarding binocularity, the BF score also experienced a significant improvement (*p* < 0.001), with a mean change of 1.55 with therapy. Figure 2 shows the change in the distribution of measurements of near stereopsis with therapy. As shown, the percentage of patients with no measurable stereopsis was 65.8% before therapy and decreased significantly to 11.8% after the training (*p* < 0.001). Furthermore, 99 patients (61.5%) did not report flat fusion with the 4-dot Worth test before visual training, whereas this number decreased to 15 (6.3%) after finishing the therapy (*p* < 0.001). The change achieved in BCVA was significantly correlated with spherical equivalent (r = −0.256, *p* = 0.001), baseline BCVA in the non-dominant eye (r = −0.584, *p* < 0.001) (Figure 3), baseline interocular difference in BCVA (r = −0.438, *p* < 0.001), and baseline BF score (r = −0.510, *p* < 0.001) (Figure 4).

### 3.2. Analysis of the Anisometropic Amblyopia Group

**Figure 5:**
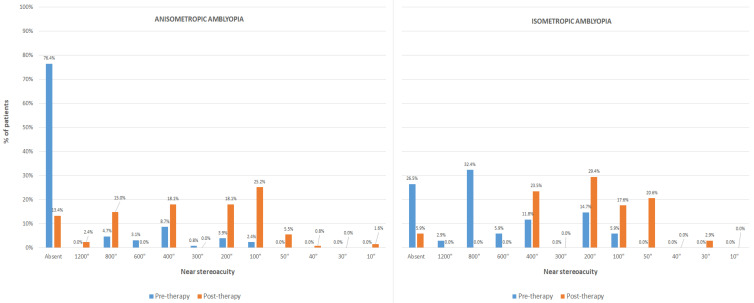
Distribution of the pre-and post-therapy measurements of near stereoacuity in the groups of anisometropic and isometropic amblyopia.

In this group of amblyopic patients, a significant improvement was also observed in the BCVA of the non-dominant eye (*p* < 0.001), with a significant improvement associated in BF score (*p* < 0.001) (Table 1). However, the dominant eye did not experience a significant change (*p* = 0.180). A total of 92 patients (72.4%) did not report flat fusion with the 4-dot Worth test before visual training, whereas this number decreased to 15 (11.8%) after finishing the therapy (*p* < 0.001). Concerning stereopsis, the number of patients without measurable stereopsis decreased significantly from 97 (76.4%) before therapy to 17 (13.4%) after therapy (*p* < 0.001). Figure 5 shows the change in the distribution of measurements of near stereopsis with therapy in this group. Finally, the change achieved in BCVA in this group was found to be significantly correlated with spherical equivalent (r = −0.257, *p* = 0.004), baseline BCVA in the non-dominant eye (r = −0.564, *p* < 0.001), baseline interocular difference in BCVA (r = −0.566, *p* < 0.001), and baseline BF score (r = −0.539, *p* < 0.001).

### 3.3. Analysis of the Isometropic Amblyopia Group

In this group, the improvement in BCVA was statistically significant in both non-dominant and dominant eyes (*p* < 0.001), with a significant improvement associated in BF score (*p* < 0.001) (Table 1). In addition, significant reductions were found with therapy in the percentage of patients without flat fusion (7 patients 20.6% vs. 0 patients 0.0%, *p* < 0.001) and the percentage of patients without measurable stereopsis (9 patients 26.5% vs. 2 patients 5.9%, *p* = 0.016) (Figure 5). Concerning the correlation of the visual change achieved with therapy in the non-dominant eye with baseline data, significant correlation of the change achieved in BCVA was found with baseline BCVA in the non-dominant eye (r = −0.635, *p* < 0.001) and BF score (r = −0.451, *p* < 0.001).

## 4. Discussion

Dichoptic visual training has been demonstrated as useful for improving the visual acuity and binocular function in different types of amblyopia [7,8,9,10,11,12,13,14,15,16,17,18,19]. This training is able to generate some neural changes, such as an evolution to a non-effort pattern of the neural activity in the frontal, parietal, and occipital lobes [26]. These changes allow overcoming some of the alterations leading to interocular suppression and consequently to a less developed visual function in the amblyopic eye [27,28,29,30]. Li et al. [29] demonstrated that interocular suppression plays a key role in the visual deficits associated with anisometropic amblyopia. Indeed, it was shown to be significantly correlated in amblyopic eyes with interocular visual acuity differences, the visual acuity of amblyopic eye, and the stereoacuity at both near and distance [31]. This interocular suppression shares a common suppression mechanism at the early stage in the pathway (e.g., striate cortex), but may have additional extra-striate contributions affecting both dorsal and ventral streams differentially [28]. The aim of the current study was to evaluate the efficacy of a new digital platform allowing online visual training in a dichoptic environment in two different groups of amblyopes, isometropic and anisometropic.

In the whole sample (including both isometropic and anisometropic amblyopes), the performance of the visual training with the digital platform evaluated during 6 weeks led to a significant improvement of the BCVA of the non-dominant eye, with mean and median changes of 0.39 and 0.42 logMAR, respectively. This supposes a change of around 4 logMAR lines on average in the whole sample analysed, which is significantly larger than those reported in previous experiences with dichoptic training [7,8,9,10,11,12,13,14,15,16,17,18,19,20,21]. Huang et al. [9] reported a 3-month improvement of BCVA in the non-dominant eye of 0.32 ± 0.15 logMAR in a sample of children (7–10 years) with bilateral anisometropic, strabismic or mixed amblyopia no longer responsive to occlusion therapy that were treated with dichoptic visual training. Mezad–Koursh et al. [15] reported a mean improvement in non-dominant eye BCVA of 0.28 log MAR in a sample of children aged from 4 to 8 years (anisometropic, strabismic or mixed amblyopia) after watching dichoptic animated videos at home using a specific device for 60 min 6 days a week, achieving a compliance rate of 88 ± 16%. Likewise, Bossi et al. [17] found in 2017 a mean improvement in BCVA in the amblyopic eye (anisometropic, strabismic or mixed) of 0.27 ± 0.22 logMAR in a sample of children from 3 to 11 years with a compliance rate with the dichoptic training of 89.4 ± 24.2%. The rest of the studies evaluating the results of dichoptic training in amblyopia reported lower improvements in the level of BCVA of the non-dominant eye after therapy [7,8,10,11,12,13,14,16,18,19,20,21]. There are many factors that can contribute to the significant differences among studies in terms of BCVA and stereoacuity gain, such as the age range of the participants of the study, the inclusion of adults mixed with children in some samples, differences in the procedure for measuring the level of BCVA or stereoacuity, the type of stimuli or the level of dichoptic dissociation during therapy, the type of interaction of the patient with the dichoptic environment used for the training (passive or active), differences in terms of follow-up and compliance rate, and the combination of different types of amblyopes. It should be remarked that anisometropic and strabismic amblyopia have different clinical behaviour and prognosis, and are even associated with different neural alterations [32,33,34]. To our knowledge, this is the first series evaluating the impact of a dichoptic therapy in exclusively refractive amblyopic eyes.

In our whole sample, the change achieved with treatment in the BCVA of the non-dominant eye was inversely correlated with baseline BCVA in the non-dominant eye, baseline interocular difference in BCVA, and baseline BF score. This means that more improvement was achieved with the dichoptic treatment performed with the digital platform evaluated in those cases with worse BCVA in the non-dominant eye at baseline, more interocular difference BCVA (more level of amblyopia), and poorer binocular function. This is consistent with the results of previous series combining in the same sample refractive and strabismic amblyopic cases [12,14]. Liu et al. [12] found that the BCVA improvement achieved after dichoptic training with Gabor patches was dependent on pre-training BCVA and that the stereoacuity gain was significantly correlated with the pretraining interocular BCVA difference. Birch et al. [14] found that more improvement in BCVA was achieved when using contrast-rebalanced dichoptic movies on a passive 3D display for the treatment of refractive or strabismic amblyopia in those cases with worse BCVA in the non-dominant eye at baseline (severe amblyopia).

Concerning stereopsis and binocularity, a composite score, the binocular function score, was used in the current analysis as this gives a more accurate estimate to characterize the binocular function, considering not only those cases in which stereopsis was measurable, but also those cases with suppression and those with flat fusion but without measurable stereopsis [25]. Using this approach, a significant improvement in BF score was found in the whole sample after dichoptic treatment, with a change of the percentage of patients without measurable stereopsis from 65.8% to 11.8% with therapy. Some previous series have reported significant changes in stereoacuity after dichoptic therapy in mixed samples of refractive and strabismic amblyopia [9,11,13,16]. Pang et al. [13] found in a sample of refractive, strabismic or mixed amblyopic patients (8 to 51 years) a mean change in stereoacuity of 0.40 log arcsec after watching contrast-balanced dichoptic videos for 6 weeks. Likewise, Huang et al. [9] obtained a significant change of stereoacuity from a baseline value of 190.00 ± 163.34 arc sec to a value of 85.00 ± 61.24 arc sec after 3 months of dichoptic training in a sample of children (7–10 years) with bilateral amblyopia. However, all these studies considered only those patients with measurable stereopsis, not considering the changes occurring in those patients with a poorer binocular function. In our sample, with the BF score, all cases were considered, including changes from not measurable to measurable stereopsis. From our perspective, an improvement in stereoacuity was possible in most of patients as they possibly developed it in the early months of their lives, but the refractive error and the anisometropia did not allow them to maintain this visual ability [35]. No cases of amblyopia due to congenital esotropia or deprivation, for which the achievement of some level of stereoacuity seems improbable, were included in this sample.

Besides the analysis of the whole sample, additional sub-analyses were performed in the subgroups of patients with anisometropic and isometropic amblyopia. In both subgroups, significant improvements were found in BCVA of the non-dominant eye as well as in BF score, confirming the efficacy of the dichoptic therapy in both types of amblyopia. However, as could be expected, a significant improvement was also observed in the BCVA of the dominant eye in the isometropic amblyopia subgroup, as patients from these groups had bilateral amblyopia, with a BCVA susceptible of being improved in both eyes. Therefore, despite both eyes being affected, significant improvements can be achieved with dichoptic training in isometropic bilateral amblyopia. This is consistent with the results of Huang et al. [9] who demonstrated that dichoptic therapy induced significant changes in BCVA and stereoacuity in patients with bilateral amblyopia no longer responsive to patching. Likewise, in both subgroups, the change in BCVA in the non-dominant eye was inversely correlated with the baseline BCVA value in such eyes and the BF score, as happened in the analysis with the whole sample. In the anisometropic subgroup, this change was also significantly correlated with the interocular difference in BCVA.

This study has some limitations that should be acknowledged. First, the retrospective nature of the study can be considered as a limitation, but the same protocol was followed for all patients, with all patients having a complete pre- and post-training visual examination using the same measurement methods and tests. Second, there was no control study or placebo group showing the superiority of the outcomes in the study compared to this control sample. Future randomized controlled clinical trials should be conducted to confirm this issue with the dichoptic digital platform evaluated. In any case, several previous trials have demonstrated the superiority of dichoptic therapy over placebo or control groups [8,13,15,19]. Finally, changes in other variables characterizing the visual function with the digital platform analysed should be evaluated such as contrast sensitivity, saccadic performance or fixation pattern.

## 5. Conclusions

In conclusion, the use of the dichoptic therapy with the Bynocs platform allows an effective restoration of visual acuity and binocular function in children with anisometropic and isometropic amblyopia. More visual improvement is expected with this type of treatment in those eyes with worse baseline BCVA and binocular function. All these results should be confirmed in future placebo-controlled randomized clinical trials.

## Figures and Tables

**Table 1:** Summary of the main outcomes obtained in the whole sample as well as in the anisometropic and isometropic groups. Abbreviations: SD, standard deviation; BCVA, best corrected visual acuity; BF, binocular function.

Mean (SD) Median (Range)	Pre-Therapy	Post-Therapy	*p*-Value
Whole sample			
Non-dominant BCVA	0.58 (0.22)	0.19 (0.19)	<0.001
	0.52 (0.20 to 1.10)	0.10 (0.00 to 0.70)	-
Dominant BCVA	0.11 (0.22)	0.03 (0.09)	<0.001
	0.00 (0.00 to 1.10)	0.00 (0.00 to 0.60)	-
BF	4.14 (1.14)	2.59 (0.91)	<0.001
	5.00 (2.00 to 5.00)	2.30 (1.00 to 5.00)	-
Anisometropic group			
Non-dominant BCVA	0.59 (0.23)	0.20 (0.20)	<0.001
	0.54 (0.20 to 1.10)	0.20 (0.00 to 0.70)	-
Dominant BCVA	0.00 (0.02)	0.00 (0.00)	0.18
	0.00 (0.00 to 0.17)	0.00 (0.00 to 0.00)	-
BF	4.39 (1.04)	2.67 (0.97)	<0.001
	5.00 (2.00 to 5.00)	2.30 (1.00 to 5.00)	-
Isometropic group BCVA			
Non-dominant BCVA	0.51 (0.19)	0.14 (0.15)	<0.001
	0.50 (0.24 to 1.10)	0.10 (0.00 to 0.60)	-
Dominant BCVA	0.49 (0.19)	0.15 (0.15)	<0.001
	0.49 (0.22 to 1.10)	0.15 (0.00 to 0.60)	-
BF	3.22 (1.01)	2.27 (0.56)	<0.001
	2.90 (2.00 to 5.00)	2.30 (1.48 to 4.00)	-

## Data Availability

Not applicable.
